# Editorial for “Mechanical Behavior of Concrete Materials and Structures: Experimental Evidence and Analytical Models (Volume II)”

**DOI:** 10.3390/ma18030584

**Published:** 2025-01-27

**Authors:** Dario De Domenico, Luís Filipe Almeida Bernardo

**Affiliations:** 1 Department of Engineering, University of Messina, 98166 Messina, Italy; 2 GeoBioTec, Department of Civil Engineering and Architecture, University of Beira Interior, 6201-001 Covilha, Portugal; lfb@ubi.pt

Concrete represents one of the most popular materials employed in the civil engineering sector due to its flexibility and adaptability to multiple applications, ranging from non-structural elements and partition walls to structural elements and load-bearing members. However, various applications require specific characteristics of concrete and motivate the development of new mix designs, additives, advanced materials and construction practices, which widen the scope of research on concrete engineering in general. This is the reason why the mechanical behavior of both concrete and reinforced concrete (RC) materials and structures still represents a primary research theme addressed by several researchers by means of various approaches, namely experimental, analytical and numerical approaches. These contributions investigate the description of the mechanical behavior of concrete materials and structures, exhibited under both serviceability and ultimate load conditions. Many complex phenomena are involved in the definition of such mechanical behavior, including but not limited to tensile cracking, compression crushing, strain softening, interaction between aggregates and a matrix, interaction between concrete and reinforcement, stiffness degradation, energy dissipation and ductility exhibited under cycling loading, etc.

Following these research motivations and a previous successful Special Issue on the topic with the authors as guest editors [1], this Special Issue, which constitutes the Volume II of the previous referred one, presents 10 papers focused on the mechanical behavior of concrete materials and structures, including both experimental findings and theoretical/numerical analyses using both conventional, code-based, and advanced methodologies. In the editors’ opinion, all the articles present undisputable scientific novelty and contribute to the understanding of the mechanical behavior of concrete materials and structures. The auspice of the editor is that this article collection can further contribute to the ever-growing research on concrete materials and structures, with the ultimate goal to realize concrete constructions that are reliable, sustainable and efficient from a structural standpoint.

Among the contributions, two papers focused on experimental findings of both concrete and RC structures. Due to the key role of shrinkage in structural serviceability analyses of RC structures, Bachofner et al. [2] presented the experimental results from long-term shrinkage measurements on monitored large-scale prismatic concrete specimens with varying cross-sectional areas, exposed to real environment conditions. The influence of seasonal environmental conditions was accounted for. The measured shrinkage strains were presented and discussed, and compared with shrinkage models proposed by international organizations. Poor agreement was observed between experimental and predicted values from the models, namely for the larger-scale models under variable environmental conditions. In addition, different shrinkage behaviors were observed between specimens with different production dates. This contribution provided new experimental data on the shrinkage behavior of concrete, which can contribute to the improvement of novel or refined shrinkage models. Harald Schuler [3] presented experimental results on the sliding shear failure of basement-clamped reinforced concrete shear walls, tested under cyclic loading. The experimental results of three test specimens, with different boundary conditions and aspect ratios, including construction joints, were presented and discussed, including the flexure–sliding interaction. This contribution provided quantitative information on the effects of sliding shear on the failure, considering the flexure–sliding interaction and the deformation state in the clamping zone. In addition, a simple estimate was proposed to predict the occurrence of sliding shear failure in this type of RC structure, which could help for the design of RC structures located in regions prone to earthquakes.

Two contributions on concrete focused on both experimental and analytical/numerical perspectives. Ji et al. [4] presented an experimental study on the failure mechanism of steel fiber-reinforced concrete (SFRC) samples, with different steel fiber volume contents and different environmental erosion conditions. The authors compared the micro-structure and the obtained axial bearing capacity deterioration laws. In addition, a grey correlation degree of axial compressive strength was analyzed. Among the findings, it was observed that steel fibers can effectively improve the concrete axial bearing capacity. However, different responses were observed under the various erosion conditions studied, with the freeze–thaw cycle and sulfuric acid environments being the conditions with the most significant impact. In addition, a durability equation related to the fiber mixture ratio and strength was presented, based on the obtained experimental data and a numerical simulation method based on the used statistical analysis technique. This contribution provided experimental data and a theoretical basis for predicting the performance deterioration of SFRC under different environmental erosion conditions. Sun et al. [5] presented an experimental study on the axial tensile strain rate effect on concrete based on both experimental tests, through axial tension tests, and numerical simulations, via the linear parallel bond model. The aim was to study the sensitivity of aggregates, mortar and interface transition zone (ITZ) on the axial tensile strain rate of concrete. Among several findings, it was observed that the rate of sensitivity of the ITZ is the strongest and that the low tensile strength of the ITZ leads to its failure in concrete. Findings on the influence of the strain rate in several concrete specimens’ mechanical properties, both experimental and numerical, were also presented and discussed. The findings from this research can contribute to concrete engineering in the range of intermediate strain rate.

The fracture behavior of concrete is still one of the most complex phenomena to simulate due to the heterogeneous microstructure and interaction between aggregates and a matrix. Hence, among the contributions, a paper of this Special Issue focused on the mesoscopic analysis of aggregates, both rounded and hybrid, in recycled rubber concrete (RRC): Kamel et al. [6] proposed a novel and sophisticated hybrid random aggregate model for RRC, by combining convex polygon aggregates and rounded rubber co-casting schemes, with tools developed with computational codes. Based on the base force element method (BFEM) of the complementary energy principle, numerical analyses were performed to study the tensile and compressive behavior of RRC. The results showed that the ITZ around the rubber is the weakest component, where cracks form and propagate, allowing us to understand the RRC mechanism failure from a meso-damage analysis perspective. It was found that the hybrid aggregate model outperformed the rounded aggregate model. The proposed model was validated through experimental findings. The findings from this research can contribute to RRC engineering.

In recent years, an increasing number of researchers have advanced the use of machine learning techniques for predicting the mechanical properties of concrete and RC structures based on training and validating sets of data, allowing us to develop reliable predictive models without investing much effort, time, or resources in experimentation programs. Among the four contributions belonging to this topic, two focused on the strength of special concretes and the other two focused on the strength of RC structures.

For estimating the compressive strength of ecofriendly concrete, Alhakeem et al. [7] used a hybrid model of a gradient boosting regression tree (GBRT) with the grid search cross-validation (GSC-GBRT) optimization technique. To this end, a dataset including the compressive strength of eco-friendly concrete was obtained from the literature. The input variables were the water/binder ratio, curing time, recycled aggregate percentage from the total aggregate in the mixture, ground granulated blast-furnace slag material percentage from the total binder used in the mixture, and superplasticizer. After the training stage, the accuracy of the model was studied based on statistical indicators between the observed and predicted strengths. It was shown that, when compared to the default GBRT model, the GridSearchCV approach is more robust and reliable. In addition, the Shapley Additive Explanation (SHAP) approach was used to explain the significance and contribution of the input factors that affect the compressive strength. Li et al. [8] estimated the compressive strength of SFRC employing ensemble approaches (SVR AdaBoost and SVR bagging) and one individual technique (support vector regression (SVR)). The dataset was gathered from previous publications. In total, 10 input parameters were considered, namely cement, water, sand, coarse aggregate, superplasticizer, silica fume, fly ash, steel fiber, fiber length, and fiber diameter. To evaluate the efficiency of each used approach, statistical indicators and k-fold cross validation were used. In addition, a sensitivity technique was applied to assess the influence of the input parameters on the predicted results. The results showed that the SVR AdaBoost method was the most precise and performant model to predict the compressive strength of SFRC.

Ahmad et al. [9] performed a comparative analysis on the structural response of RC beams without stirrups (first crack load, flexural strength, and shear strength) by using a conventional model based on the ACI building code and another approach using artificial neural networks (ANNs). For this, a dataset comprising 110 samples was collected from the literature and used to train a multilayer backpropagation neural network. The input parameters were width and effective depth of the beam, shear span to effective depth ratio, steel reinforcement ratio, Whitney block compressive area, steel yield strength, concrete compressive strength, and span of the simply supported beam. Based on a statistical evaluation, it was found that the ANN model produced results that closely align with those obtained from the conventional model, showing the potential of ANNs to be used as reliable models for the prediction of the response of RC beams without stirrups. For estimating the punching shear strength of fiber-reinforced polymer (FRP) RC flat slabs, Abood et al. [10] employed gradient boosted regression tree (GBRT) models. For this, a database of 238 sets of experimental results for FRP-reinforced concrete slabs was compiled from the literature and used as training and validating sets. The input parameters were cross-section area of the column, perimeters for the critical section in the slab, effective depth of the slab, compressive strength of concrete, steel reinforcement ratio, and modulus of elasticity of the steel and FRP rebars. From the obtained results, and based on statistical indicators, it was found that the GBRT model achieved the highest prediction accuracy, when compared to other studied models (k-nearest neighbors (KNNs) and Lasso models). In addition, the Shapley Additive Explanation (SHAP) method was used to provide insights on the contribution of each input parameter to the prediction of the punching shear strength from the GBRT model. The developed GBRT model constitutes a reliable alternative model to existing empirical models and design provisions that continue to exhibit significant bias and dispersion to predict the punching shear strength of FRP-reinforced concrete slabs.

In recent years, the durability of existing RC structures has been regarded as a crucial performance requirement. One of the most serious factors negatively affecting the durability of concrete structures is the corrosion of steel reinforcement. The paper by Syll and Kanakubo [11] presents a systematic literature review to explore experimental methods, outcomes, and trends on the corrosion of the reinforcement, and its impact on the bond capacity between concrete and rebars. Based on an extensive literature review, the review paper discusses the degradation mechanism of bond strength affected by corrosion, and the key factors affecting the bond strength degradation, such as concrete cover, concrete strength, and stirrups. Although empirical, theoretical, and numerical predictive models have been proposed in previous studies, the authors emphasize that such models are still limited due to discrepancies caused by different testing methods in the evaluation of the effect of corrosion on the bond strength. Hence, new guidelines were proposed to build a practical and reliable model to assess the bond strength deterioration of corroded RC.

## References

[1] De Domenico D., Bernardo L.F.A. (2022). Mechanical Behavior of Concrete Materials and Structures: Experimental Evidence and Analytical Models.

[2] Bachofner W., Suza D., Müller H.S., Kollegger J. (2023). Long-Term Shrinkage Measurements on Large-Scale Specimens Exposed to Real Environmental Conditions. Materials.

[3] Schuler H. (2024). Sliding Shear Failure of Basement-Clamped Reinforced Concrete Shear Walls. Materials.

[4] Ji Y., Xu W., Sun Y., Ma Y., He Q., Xing Z. (2022). Grey Correlation Analysis of the Durability of Steel Fiber-Reinforced Concrete under Environmental Action. Materials.

[5] Sun B., Chen R., Ping Y., Zhu Z., Wu N. (2022). Study on Axial Tensile Strain Rate Effect on Concrete Based on Experimental Investigation and Numerical Simulation. Materials.

[6] Kamel M.M.A., Fu Y., Feng X., Peng Y. (2023). Mesoscopic Analysis of Rounded and Hybrid Aggregates in Recycled Rubber Concrete. Materials.

[7] Alhakeem Z.M., Jebur Y.M., Henedy S.N., Imran H., Bernardo L.F.A., Hussein H.M. (2022). Prediction of Ecofriendly Concrete Compressive Strength Using Gradient Boosting Regression Tree Combined with GridSearchCV Hyperparameter-Optimization Techniques. Materials.

[8] Li Y., Zhang Q., Kamiński P., Deifalla A.F., Sufian M., Dyczko A., Kahla N.B., Atig M. (2022). Compressive Strength of Steel Fiber-Reinforced Concrete Employing Supervised Machine Learning Techniques. Materials.

[9] Ahmad M.M., Elahi A., Barbhuiya S. (2023). Comparative Analysis of Reinforced Concrete Beam Behaviour: Conventional Model vs. Artificial Neural Network Predictions. Materials.

[10] Abood E.A., Abdallah M.H., Alsaadi M., Imran H., Bernardo L.F.A., De Domenico D., Henedy S.N. (2024). Machine Learning- Based Prediction Models for Punching Shear Strength of Fiber-Reinforced Polymer Reinforced Concrete Slabs Using a Gradient-Boosted Regression Tree. Materials.

[11] Syll A.S., Kanakubo T. (2022). Impact of Corrosion on the Bond Strength between Concrete and Rebar: A Systematic Review. Materials.

